# Evaluation of Calcium Aluminate Slags and Pig Irons Produced from the Smelting-Reduction of Diasporic Bauxite

**DOI:** 10.3390/ma14247740

**Published:** 2021-12-15

**Authors:** Adamantia Lazou, Leiv Kolbeinsen, Jafar Safarian

**Affiliations:** Department of Materials Science and Engineering, Norwegian University of Science and Technology, 7034 Trondheim, Norway; leiv.kolbeinsen@ntnu.no (L.K.); jafar.safarian@ntnu.no (J.S.)

**Keywords:** Pedersen process, alumina production, bauxite, smelting reduction, calcium aluminate slags, iron

## Abstract

This work evaluates the characteristics of calcium aluminate slag and pig iron samples obtained from the smelting of calcined and reduced diasporic bauxite ore. The study is conducted in the Pedersen process framework, which is a method to produce alumina from low-grade resources. Parameters such as the effect of crucible type, lime addition, and atmospheric conditions are studied considering the characteristics of the product pig irons and calcium aluminate slags for further uses. The behavior of the bauxite and distribution of the species between slag and metal was assessed based on the applied analytical techniques and thermodynamic calculations. Iron was reduced and separated from the slags in the presence of carbon (graphite crucible) for both the reduced and calcined bauxite. Si and Ti were mainly concentrated in the slags. Iron was separated from the slag in the absence of carbon (alumina crucible) for the H_2_-reduced bauxite. The results show that slags with increased lime additions are composed mainly of 5CaO.Al_2_O_3_ and CaO.Al_2_O_3_, that are considered highly leachable compounds. An optimum CaO/Al_2_O_3_ mass ratio of 1.12 was suggested. The presence of O_2_ and/or OH^-^ in the furnace atmosphere will result in the formation of 12CaO.7Al_2_O_3_.

## 1. Introduction

Considering the depleting tendency of the high-grade ore deposits, alternative processes are necessary to be developed and utilize the available resources efficiently and holistically. Aluminum metal, which finds broad applications due to its properties, is produced mainly through metallurgical grade alumina. The Bayer process is the current dominant commercial method used to produce alumina. Even though the Bayer process has been adapted for years, it has environmental challenges—the main being the formation of the Bauxite Residue (BR) [1,2,3]. Another limitation relates with the characteristics of the utilized ore since they will affect the overall process [4]. Low Al_2_O_3_/Fe_2_O_3_ ratio in the bauxite ore is prohibited in the Bayer process since higher amount of the generated BR will be produced [5]. As such, the development of processes that can utilize lower grade alumina-containing sources is essential.

**Figure 1:**
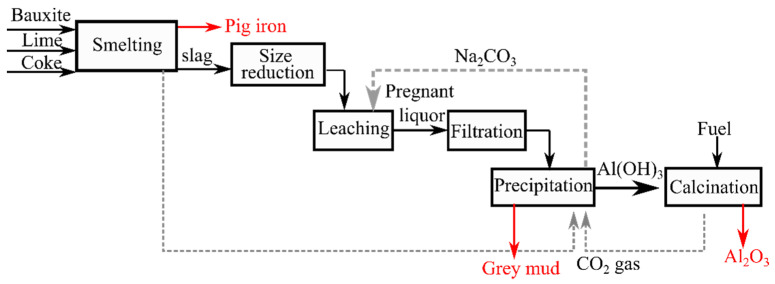
A simplified process flow of the Pedersen process, the stream by-products are highlighted in red color.

The Pedersen process was commercialized and is a high temperature (molten state) process that can separate iron and calcium aluminate slag [5]. The method is regarded to be more material efficient and flexible in terms of the raw materials qualification to produce alumina as it can holistically utilize the bauxite or other alumina sources and avoid the production of BR [5]. Further details on the process can be found in the available bibliographic sources [5,6,7]. Significant research has been conducted recently in the framework of the EnsureAl EU project for modernizing the Pedersen process [8,9,10,11,12,13,14,15]. As an example in our earlier work, the partial replacement of coke with charcoal was studied and it was found to ease the smelting process [16]. The simplified flowsheet of the process can be seen in Figure 1. 

The slag mineralogy and other process parameters will play a key role in the characteristics of the product slags and the subsequent leaching efficiency of the alumina. Further details on the slag characteristics and the calcium aluminate (C-A) phases have been reported in earlier publications [9,17,18,19,20,21,22]. The 12CaO.7Al_2_O_3_ (C_12_A_7_) and the metastable 5CaO.3Al_2_O_3_ (C_5_A_3_) phases, have similar chemistry and are classified as the most leachable C-A compounds. Their stabilization depends on the atmospheric conditions and/or in the presence of other anions in the furnace atmosphere [17,20,21,22]. The C_5_A_3_ is an ordered layered structure with crystal density 3.03 g/cm^3^ [22] while the C_12_A_7_ is a disordered clathrate with crystal density of 2.68 g/cm^3^. 

In our earlier articles, emphasis was given in the valorization of the bauxite residue as a feed material for the Pedersen process [17,23,24]. Adapting a similar research strategy, this work focuses on the smelting reduction of diasporic bauxite ore. Preliminary experiments [16] were first conducted in a bigger scale (>0.4 kg bauxite), offering the basis for the present approach that seeks to investigate more the effect of process parameters in the final products. Earlier works mainly focused on the carbothermic smelting reduction or semi-smelting reduction of iron-rich bauxites [9,25] and usual parameters are the reduction temperature, carbon to iron ratio, and slag basicity. The gas reduction of bauxite has been poorly reported, and its consideration could offer significant advantages in the overall process. When the Pedersen process was commercialized, the environmental issues that are currently demanding were not that crucial. Among others, the decrease of the CO_2_ gas emissions from the smelting furnace is of importance. Recent technological advantages can be used to overcome such obstacles. In particular, the pre-reduction of bauxite by H_2_ gas can decrease the demand in carbon-based reductants and herein decrease the process CO_2_ gas emission. As such, the H_2_-reduction of bauxite prior to its smelting is suggested in the present work and considered as a novel approach. The present study seeks to exploit and optimize the conditions to achieve highly leachable slag by analyzing the effect of the studied parameters in the product phases. The products were analyzed for their chemical, mineralogical, and microstructure characteristics. In addition, the characteristics of the feedstock bauxite samples (calcined and H_2_-reduced) and the different smelting conditions on the characteristics of the products are evaluated based on the theoretical and experimental results.

## 2. Materials and Methods

This section describes the experimental procedure that was followed in the present research.

### 2.1. Preparation and Analytical Techniques

Bauxite ore was provided by Mytilineos AS Metallurgy Unit, Viotia, Greece (former Aluminium of Greece, AoG). Quicklime was used as flux, and it was provided from Franzefoss Minerals AS. The ore with a particle size of 2.4–4.8 mm was calcined at 900 °C in a muffle furnace for 3 h. The sample is called CB. Representative CB and quicklime samples were then dried at 100 °C in a static furnace for 24 h prior any further treatment. Part of the utilized CB (148 g) was reduced by 50 vol.-%H_2_ and 50 vol.-%Ar mixture to obtain reduced bauxite, RB, with 2 Nl/min total flow rate at 900 °C for 120 min, using a thermogravimetric (TGA) technique. Details of the equipment can be found in our previous publication [26]. The main purpose being the reduction of the iron oxides in bauxite to metallic iron prior to the smelting and the subsequent comparison with the smelting-reduction of the calcined bauxite. The reduced and calcined samples were further crushed to powder to increase the contact and surface area for the reactions during the heating and smelting. The quicklime was received in powder form. Mixtures of lime with CB or RB samples were prepared in different analogies. The CaO additions were based on mass balance calculations according to the characteristics of the raw materials and to achieve 12CaO.7Al_2_O_3_ (C_12_A_7_), CaO.Al_2_O_3_ (CA), CaO.TiO_2_ (CT), and 2CaO.SiO_2_ (C_2_S) phases in the slags. The studied range was selected to obtain phases that are considered leachable. The experimental design and the corresponding changes in the slag phases were further supported from the theoretical calculations. 

The particle size distribution of the input materials was measured with laser particle size analyzer, Partica LA-950 Horiba, Kyoto, Japan. The chemical composition of the raw and product samples was determined through X-ray Fluorescence (XRF) technique, Thermo Fisher, Switzerland. The mineralogical analysis was done by X-ray Diffraction (XRD) technique using a Bruker D8 A25 DaVinci^TM^, Karlsruhe, Germany, with CuKα radiation in 2θ range of 10 to 75° diffraction wavelength and 0.02 deg step size. For that purpose, the materials were crushed to fine powder and the assessment was done using the available crystallographic databases (PDF) and with the Diffract. EVA Software. Selected samples were refined for their quantitative phase analysis (Rietvelt) with the use of TOPAS software (version 5). TOPAS was originally developed by Alan Coelho in Brisbane, Australia and the version used in the present study is the commercially available edition that was developed by Bruker and Coelho. The microstructure of the raw and product materials was assessed employing Scanning Electron Microscope (SEM), using FE-SEM, Zeiss Ultra 55 LE, Carl Zeiss, Jena, Germany. The distribution of the elements was further evaluated with the use of X-ray elemental mapping and use of Energy Dispersive Spectroscopy (EDS), Bruker AXS, microanalysis GmbH, Berlin, Germany. Furthermore, JXA-8500F^TM^ Electron Probe Micro-Analyzer (EPMA) supported with Wavelength Dispersive Spectroscopy (WDS), JEOL Ltd., Tokyo, Japan, for the accurate chemical analysis of the apparent phases in the product species was employed. The composition of the obtained pig iron was done with EPMA. 

### 2.2. Smelting and Smelting-Reduction Trials

The main parameters that are studied in the smelting and smelting-reduction trials are the CaO additions, charge material (CB, RB), cooling rate, and the atmospheric conditions (Table 1). Most of the results are referred to CB due to sample availability. Most of the experiments were conducted in a close-top induction furnace, ELEKTRO-MASCHINEN, Schultze GmbH & Co. KG, Hirschhorn, West Germany. An open top induction furnace was also used, which seeks reducing conditions in the presence of air. The cooling was controlled through the decrease of furnace power to reach 1000 °C in approximately 75 min, which gives an average cooling rate of ~10 °C/min for most of the experiments in both the closed and open furnaces. To examine the effect of the moisture in atmospheric reducing conditions, the presence of higher O_2_ partial pressure were adjusted with the use of Ar-H_2_O gas in closed furnace in the presence of carbon, open top induction furnace with sample exposure to air in the presence of C, and alumina crucible in closed furnace under Ar gas, respectively. The H_2_O-containing gas was introduced through a humidifier in which steam (H_2_O of 5 vol.%) is added into the dry argon and the humidified gas is introduced into the melt. Selected experiments were repeated to ensure the reproducibility of the results. The mixtures of 35 g of CB or RB and lime in adjusted amounts were placed in two different inner (sample) crucibles, an alumina (A5, Ø50/44 -by- 75 mm) and a graphite (IG-15, Ø60/52 -by- 100 mm), which were placed inside an outer graphite crucible. The furnace chamber was evacuated and then filled up by argon. The power was then regulated to reach the target temperature. The smelting temperature was regulated to 1650 °C ± 20. The temperature was recorded with a thermocouple C, which was put in an alumina insulating tube and was placed in between the outer graphite crucible and the inner sample crucible. The $p_{O_{2}}\;$ in Table 1 was estimated with FactSage and based on a simplified slag composition (CaO, Al_2_O_3_, SiO_2_, TiO_2_, and Fe_2_O_3,_ FTlite, and FToxid databases). The amount of H_2_O was calculated assuming ideal gas behavior and based on the input amount (5 vol.%). The calculated $p_{O_{2}}\;$ is used to offer an estimation for the changes in the different scenarios.

**Figure 2:**
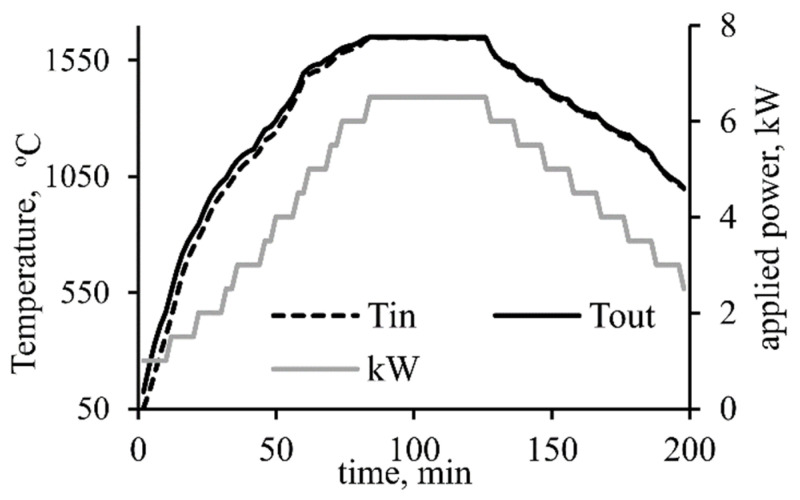
Thermocouple reading from inside (T_in_) and outside (T_out_) of the inner graphite crucible and the applied power (kW) used for heating and cooling from the closed furnace.

A typical temperature profile (heating, holding, and cooling) can be seen in Figure 2. The measured temperatures are representative of the sample crucible (inner) as seen in Figure 2. 

After the smelting-reduction trials, the crucibles were removed from the furnace, and they were cut to separate the slag and metal phases. The slag was further crushed to powder and representative samples were analyzed with XRF and XRD, while slag pieces were analyzed with SEM and EPMA techniques. The metallic samples were analyzed using the EPMA technique. 

### 2.3. Thermodynamic Equilibrium and Scheil-Gulliver Calculations

Equilibrium and Scheil-Gulliver calculations were conducted using FactSage 7.3 thermochemical software [27]. The commercial FToxid, FactPS, and FTlite databases were used. Equilibrium calculations were conducted in the smelting temperature and based on the S-CB-L1, S-CB-L3, and S-RB compositions with varying carbon additions. Scheil-Gulliver cooling calculations were conducted subsequently on the predicted liquid compositions for the phase’s distribution [27,28]. The main purpose being to support the experimental design and to evaluate the obtained results. Due to lack of thermodynamic data for the C_12_A_7_ phase in the commercial databases, a dedicated database developed in EnsureAl project was also used and the thermodynamic data were based on the available literature [29]. Nevertheless, there is a lack in the thermodynamic data for the metastable C_5_A_3_ and therefore it was not possible to be included in the calculations. 

HSC Chemistry 10 software was used [30] to provide an estimation basis for the phase composition of the slags. The species converter module was used, which allows to convert elemental analysis to species. For the output analysis, a greater weight coefficient was considered for the main phases (C_12_A_7_, C_2_S and CT), which was based on the experimental results.

## 3. Results

The obtained theoretical and experimental results will be presented as follows. 

### 3.1. Theoretical Results

**Figure 3:**
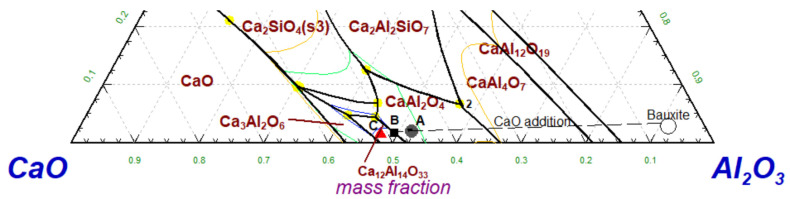
Section of the ternary phase diagram of CaO-Al_2_O_3_-SiO_2_ calculated with FactSage. The figure presents the composition of feedstock bauxite (excluding Fe and minor elements), and the point A corresponds to S-CB-L1, point B to S-CB-L2 and point C to S-CB-L3 compositions.

The composition of bauxite (ternary) is projected in the ternary phase diagram of CaO-Al_2_O_3_-SiO_2_ (Figure 3) along with the target compositions (ternary) for the S-CB-L1, S-CB-L2, and S-CB-L3 slags, points A, B, and C, respectively. Figure 3 presents the expected differences on the primary calcium aluminate phase for the different lime additions, from a thermodynamic point of view. As an example, point A is on the primary crystallization area of CA, while point C is placed in the primary area of C_12_A_7_. As such, increased lime additions are expected to enhance the formation of the leachable phases. Moreover, Figure 3 indicates that the non-leachable C_2_AS will not be favored according to the thermodynamic predictions. 

**Figure 4:**
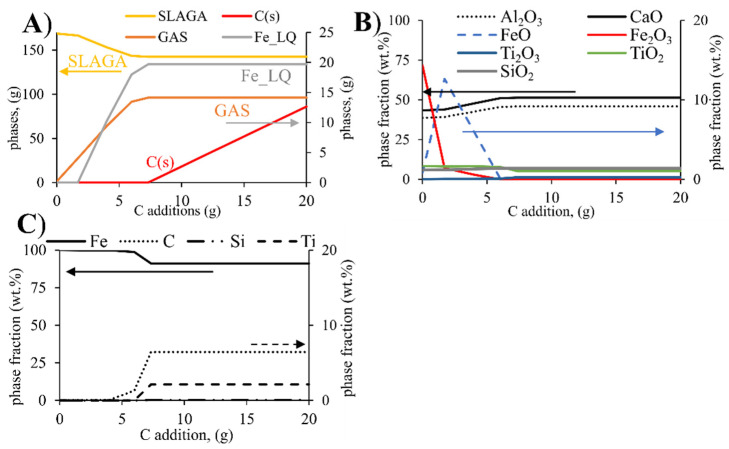
Equilibrium calculations at the smelting temperature (**A**) occurrence of different phases with varying C addition; (**B**) slag composition with varying C addition; and (**C**) metal composition with varying C addition. Secondary vertical axis (*x*-axis) displays the species with lower concentration than the major components.

Equilibrium calculations were done to examine the equilibrium of the phases, and the distribution of the chemical species between the slag and metal. Typical results are presented in Figure 4A for the S-CB-L3 composition. At the smelting temperature (1650 °C), the system is mainly composed of the liquid slag (SLAGA), liquid Fe (Fe_LQ), and stable C (s) phase. The composition of the metal and slag are presented in Figure 4B,C, respectively. The slag phase is mainly composed of Al_2_O_3_ and CaO while the metallic phase is mainly composed of Fe, as expected according to the thermodynamic stability of these species under the applied conditions. The Fe_2_O_3_ and FeO will be reduced from the slag by carbon additions. 

**Figure 5:**
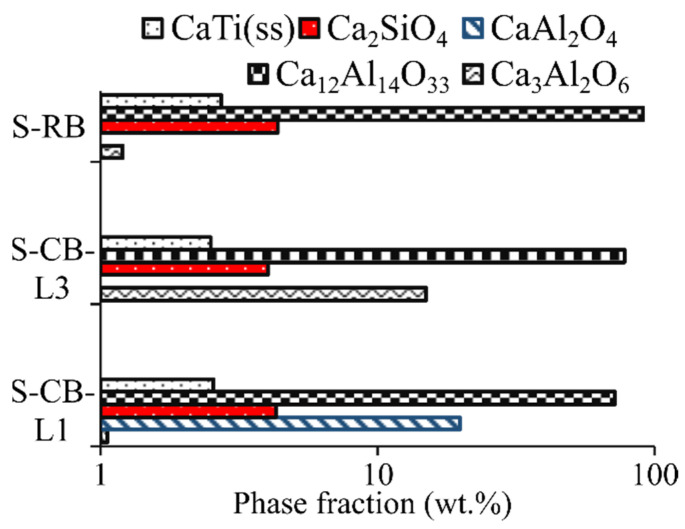
Phase distribution for S-CB-L1, S-CB-L3, and S-RB based on Scheil-Gulliver cooling calculations, *y*-axis in logarithmic scale.

For C additions higher than ~7 g (carbon saturation conditions), the iron oxides will be completely reduced to the metallic phase. The TiO_2_ will be partially reduced to Ti_2_O_3_ and for higher C concentrations (>7%) the Ti will be transferred significantly and distributed mainly to the metallic phase. It is believed that the carbon saturation conditions are closer to the experimental conditions as the graphite crucible ensures sufficient carbon. Scheil-Gulliver cooling calculations were subsequently done based on the previously calculated liquid slag compositions for low and high lime additions and are presented in Figure 5 (in logarithmic scale). For the low CaO addition case (S-CB-L1) the phases in descending order are C_12_A_7_, CA, α-C_2_S, CaTi (ss), and C_3_A. Increasing CaO (S-CB-L3 and S-RB) will result in the formation of C_12_A_7_, C_3_A, α-C_2_S, CaTi (ss). CaTi (ss) is a solid solution composed of Ca_3_Ti_2_O_7_(s)-Ca_3_Ti_2_O_6_(s). The last liquid will disappear for temperatures lower than 1306 °C. 

### 3.2. Experimental Results

The characteristics of the input materials, i.e., reduced, and calcined bauxite, and the characteristics of the products, i.e., slags, and pig irons, will be presented as follows. 

#### 3.2.1. Input Materials Characteristics

**Figure 6:**
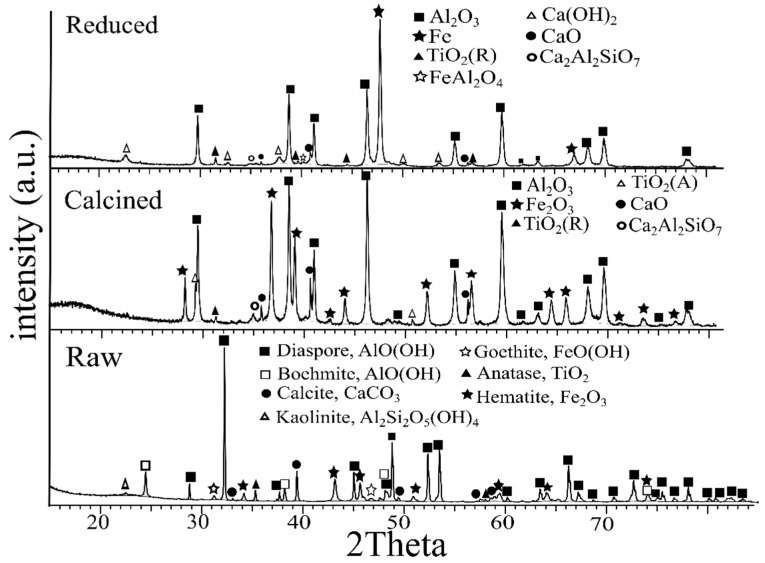
XRD analysis of raw, calcined, and H_2_-reduced bauxite.

The *D*_90_ of the dried CB, RB, and lime was found to be below 125.4 μm ± 2, 93.5 μm ± 2 and 171.2 μm ± 5, respectively. The XRD analysis results of the raw, calcined, and reduced bauxite are presented in Figure 6. The composition of the starting materials can be seen in Table 2, in dry basis.

Based on the XRD analysis of the raw bauxite, aluminum hydroxides of diaspore and boehmite were the main Al-containing phases. Iron was found in the form of hematite Fe_2_O_3_ while lower intensity peaks of goethite (FeO(OH)) were also detected. Titanium was detected as anatase (TiO_2_) and calcium as calcium carbonate (Ca(CO_3_)). The calcination at 900 °C resulted in the alternation of the aluminum hydroxides to Al_2_O_3_. CaCO_3_ was decomposed to CaO. Titanium was in the form of rutile (TiO_2_ (R)) and anatase (TiO_2_ (A)) in the CB, while in the RB was found in the form of rutile (TiO_2_). Iron was reduced almost completely to metallic iron (Fe) for the RB. Weak intensity peaks of Gehlenite (Ca_2_Al_2_SiO_7_) were detected for the CB and RB. Finally, part of the lime (CaO) was transformed in portlandite (Ca(OH)_2_), which might be due to the moisture absorption upon sample preparation for XRD. According to the XRF analysis of the CB and RB in Table 2, the calcined and reduced bauxites were composed of mostly Al and Fe, while Ca, Si, and Ti oxides have lower concentrations. In return to the XRD analysis, the iron in the RB was detected as metallic iron rather than Fe_2_O_3_. 

Therefore, the Fe_2_O_3_ content of RB that was reported from the XRF analysis has been transformed into Fe in Table 2 and for the mass balance calculations to determine the required CaO additions for the slag making process. The chemical composition was normalized correspondingly. Oxides with concentrations lower than 0.1 wt.% (V_2_O_5_, S, P_2_O_5_, and NiO) were not included in Table 2. The lime has a purity of 96.6 wt.% while the rest of the detected oxides and remaining CaCO_3_ have lower concentrations. 

#### 3.2.2. Products Characteristics

**Figure 7:**
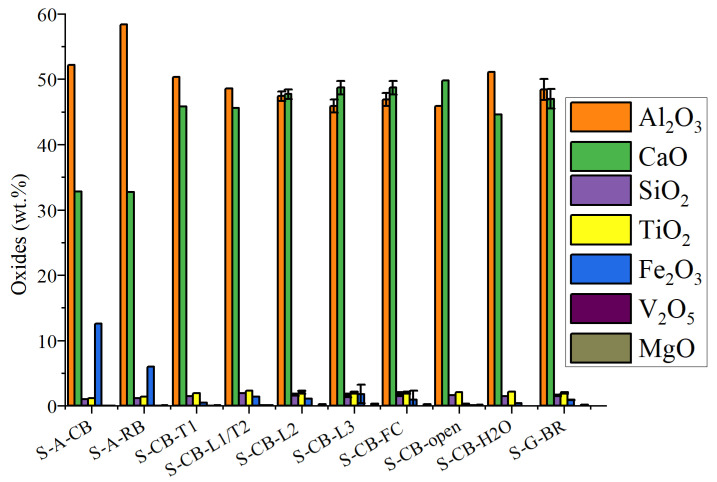
XRF analysis of slags, error bars according to repeated experiments.

Figure 7 summarizes the XRF results of the produced slags. The slags are mainly composed of CaO and Al_2_O_3_, and the iron content is low. The repeated experiments reveal a proper reproducibility, as the deviations in the chemical compositions were found to be low. The concentration of Al_2_O_3_ is significantly higher in the slags produced in Al_2_O_3_ crucibles (S-A-CB and S-A-RB) than in the slags produced in graphite crucibles. This might indicate that the slags have been contaminated from the Al_2_O_3_ crucible, while a relatively high Fe concentration was also observed most likely due to poor separation of metal from the slag. Moreover, it was observed that the mCaO/mAl_2_O_3_ ratio was lower than the target for the S-CB-H_2_O, yet in the desirable range, and this might be partially due to some inhomogeneities among the samples or due to slight fluctuations during the experiments, e.g., the temperature. For the rest of the slags, the mCaO/mAl_2_O_3_ ratio was in the desirable range as defined from the mass balance calculations.

##### Holding Time

**Figure 8:**
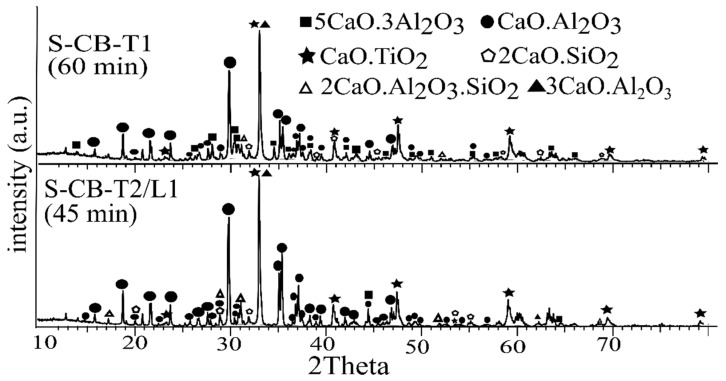
XRD analysis of the slags at different durations.

According the XRD analysis of the slags S-CB-T1 and S-CB-T2/L1 in Figure 8, the product phases were identical and as such the holding time did not significantly affect the phases at the studied temperature. CA was the main calcium-aluminate phase for both slags. The slag S-CB-T1 additionally contains C_5_A_3_, while C_2_S and CT were detected in both slags. C_3_A was also detected, but it is however challenging to precisely quantify this phase due to the overlapping with the CT phase. In general, the obtained results are also in line with the theoretical expectations (Figure 3). The difference in the intensity of the CA phase at around 35 degrees between the two samples might correlate with the predominance of this phase in sample S-CB-T2. Nevertheless, the complexity of the samples makes the exact reason difficult to claim. 

##### CaO/Al_2_O_3_ Ratio

**Figure 9:**
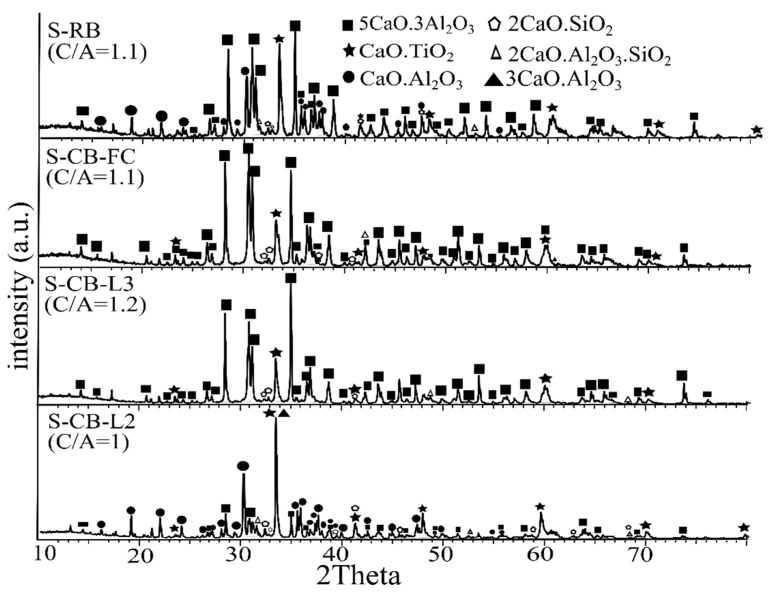
XRD analysis of the slags S-CB-L2, S-CB-L3, S-CB-FC, and S-RB produced in graphite crucibles.

Increasing the CaO/Al_2_O_3_ ratio enhances the formation of C_5_A_3_ as the main C-A containing phase according to the XRD analysis of the tests S-CB-L1 (Figure 8), S-CB-L2, and S-CB-L3 in Figure 9. In the S-CB-L2, both CA and C_3_A were seen. The peak of C_3_A overlaps with CT, which makes the precise quantification difficult. The formation of C_2_S and CT phases were common for all the slags, while low intensity peaks of C_2_AS were detected in the slags S-CB-L1 and S-CB-L2. The dominant phase of the slag S-CB-FC is the C_5_A_3_ as for the S-CB-L3 (Figure 9). As such, the increased cooling rate did not affect the product phases. However, a difference was observed in the hardness of the material upon crushing, and this should be examined further, considering its leachability. 

The slag from the H_2_-reduced bauxite (S-RB) in Figure 9 indicates that the main phase is the C_5_A_3_ as for the slag of the calcined bauxite (S-CB-L3). It is evidenced that C_5_A_3_ was the dominant C-A containing phase, while CA was also detected. Besides, CT and C_2_S were also seen. Thereafter, the slags produced from both the reduced and calcined bauxite seem to be identical and contain highly leachable phases (C_5_A_3_ and CA). It is worth to notice that during the smelting of the reduced bauxite the slag was seen to be less foamy, and this might be attributed to the less gas generation since iron was already reduced.

##### Effect of Atmosphere and Crucible

**Figure 10:**
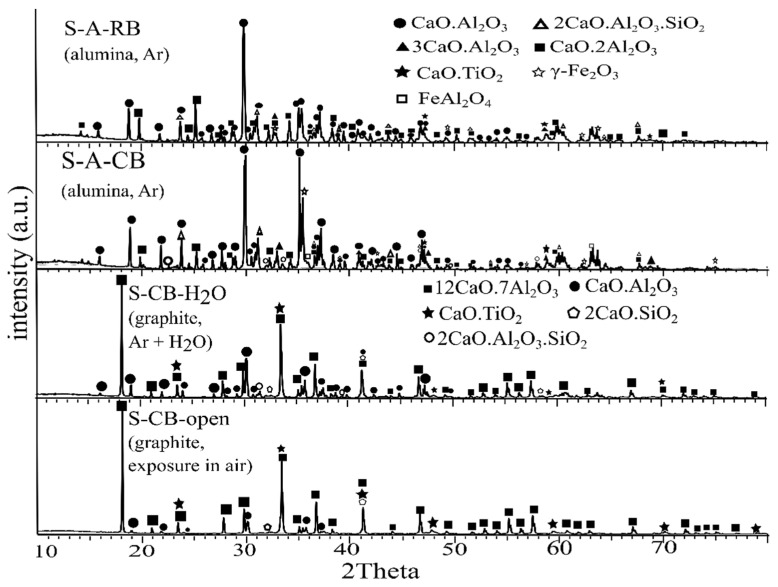
XRD analysis of the slags obtained from the studied atmospheric conditions in different crucibles.

The XRD analysis of the slags S-CB-open, S-CB-H_2_O, S-A-CB, and S-A-RB, is given in Figure 10. For the slags S-A-CB and S-A-RB apart from CA, CA_2_ was also detected, which indicates the dissolution of the crucible material into the slag, which confirms the observation made earlier according to the XRF analysis. Neither the C_5_A_3_ nor the C_12_A_7_ phases were detected in these slags. The dissolution of the alumina crucible into the slag might prohibit the stabilization of C_12_A_7_ [31] while under these experimental conditions C_5_A_3_ seems unstable. The iron was recovered in the metallic phase for the S-A-RB as was expected since iron was already reduced. The slags S-CB-open and S-CB-H_2_O were composed mainly of C_12_A_7_ and CA to a lesser extent. 

##### Phase Quantification 

Typical phase quantification results are presented in Table 3. As will be discussed below, due to the limitations of this method, the results are considered to provide a semi-quantitative estimation. 

Alumina is distributed between CA, CA_2,_ and C_2_AS for the samples smelted in alumina crucible (S-A-CB, S-A-SR). A higher amount of CA_2_ was quantified for the S-A-SR than S-A-CB due to the higher Al_2_O_3_ content of this slag (Figure 7). The silicon is distributed between the C_2_AS and C_2_S for the S-A-CB while for sample S-A-SR in the C_2_AS. The C_2_S was seen in two modifications, which might indicate a non-uniform cooling during solidification. For samples S-CB-L1, S-CB-L2, the alumina is distributed between the CA and CA_3_, and for the S-CB-L3 in C_5_A_3_. The C_2_AS and C_5_A_3_ phases could not be quantified precisely in the S-CB-L1 and S-CB-L2, respectively, most likely due to their low concentration.

Clearly, the increase in lime additions resulted in the C_5_A_3_ dominance area. The C_3_A phase improved the fitting quality of the S-CB-L1 and S-CB-L2 although its formation or more specifically its high participation in the samples is questionable according to the thermodynamic analysis presented above. It should be highlighted that the C_3_A and CT phase overlap makes the quantification challenging. In addition, the samples are composed of several phases that increase the complexity and difficulty for their quantification. Alumina is distributed between the CA and C_12_A_7_ phase for the S-CB-H_2_O and S-CB-open. For all samples Ti was distributed in the CT phase. 

**Figure 11:**
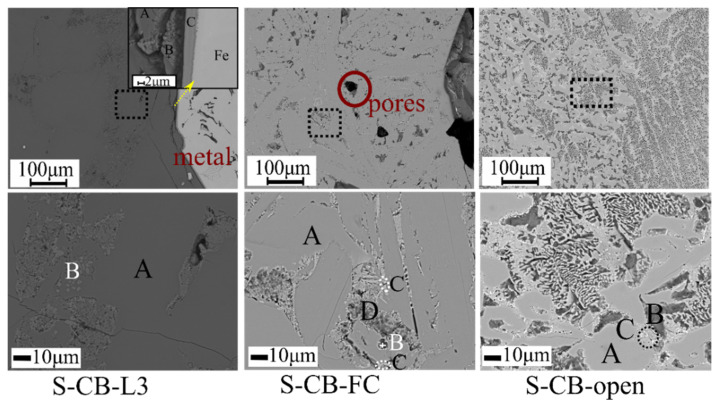
SEM backscattered electron images of S-CB-L3, S-CB-FC, and S-CB-open slags.

The SEM images of the slags S-CB-L3, S-CB-FC and S-CB-open are presented in Figure 11. The chemical composition of the marked phases based on EDS is given in Table 4. It is worth to notice that the suggested phases in Table 4 are based on the chemical compositions obtained and in correlation with the XRD analysis. 

The major phase in the S-CB-L3 is a homogeneous dark grey phase (Phase A-L3) that is mainly composed of Ca and Al. The Ca/Al ratio is approximately 0.96. Thereafter, the phase A-L3 might be attributed in the C_5_A_3_ phase detected in the XRD analysis. A brighter in appearance phase is also formed (phase B-L3) to a lesser extent and is mainly concentrated around the main phase A-L3 in a random distribution.

The phase B-L3 has high concentration of Ca and Ti, while around 12.5 at. % of Al was detected in this phase. The small size of this phase makes the precise analysis difficult. The Al content of phase B-L3 might be from the surrounding phase as a Ca-Ti-Al-O phase was not seen in the XRD analysis considering however the complexity of the obtained diffractograms. Based on other studies [43] the CaO.TiO_2_ phase might incorporate some Al (as CaTi_1−x_Al_x_O_3−δ_, 0 ≤ x ≤ 4) in its structure, which preserves the diffraction peaks of CaO.TiO_2_. A similar behavior was seen in our earlier work and the formation of this phase should be studied further [17]. The entrained Fe phase is surrounded by a phase rich in Ti, V, and C at the interface with the slag. The carbon content detected in the phase C-L3 is mainly from the C coating of the SEM sample preparation. 

The micrographs of the S-CB-FC (Figure 11) indicate that the primary phase of the slag is a light grey phase, phase A-FC. A darker grey phase B-FC and two irregulars in shape phases C-FC and D-FC were also seen to a lesser extent. Pores were observed throughout the surface of the sample while no Fe-entrains were seen. The phases B-FC, C-FC, and D-FC are mainly concentrated around the main phase A-FC in a random distribution. The size and morphology of these phases make the exact identification difficult. The phase A-FC is composed mainly of Al and Ca while Si was also detected in low concentration. The Ca/Al ratio (Table 4) is ~0.96 as for the phase A-L3 in the S-CB-L3 and might be attributed to the C_5_A_3_ phase in line with the XRD analysis. The phase B-FC contains mostly Ca, Al with Si of ~6 at. %, and this phase might be non-stoichiometric C_2_AS, even though this phase was not clearly seen in the XRD results possibly due to its low concentration. Ti was concentrated in the light grey phase C-FC. Phase D-FC has higher concentration of Ca and Al while Mg, Si, and Ti were also detected. Phase D-FC contains more than one phase based on its appearance. It might be reasonable to claim that the S-CB-FC has more complex and less pure phases (B-FC, C-FC, and D-FC phases) than the S-CB-L3 and this might be attributed in the increased cooling rate. 

The slag S-CB-open is composed mainly of a light grey phase (phase A–open) that consists of Al and Ca. In correlation with the XRD analysis, it might be attributed to the C_12_A_7_ phase. A bright phase (phase B–open) and a dark grey phase (phase C–open) of irregular shape were also detected. The phase B–open is composed of Al and Ca while some Si was also detected. The phase C–open has higher concentration of Ti, than the phases A–open and B–open. However, the size and shape of phase C-open made its analysis difficult. 

**Figure 12:**
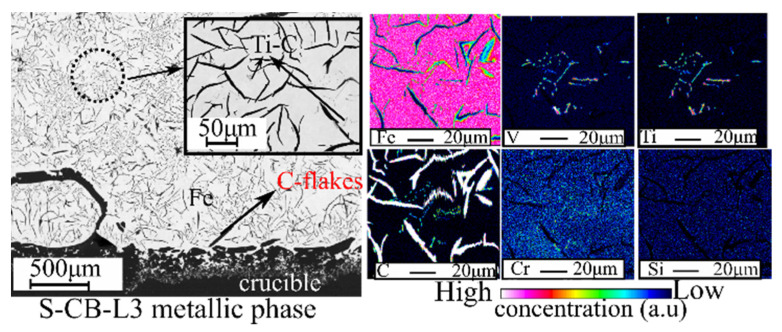
Backscattered image and X-ray elemental mapping of the metallic phase from S-CB-L3.

A typical BSE image and elemental X-ray mapping of the metallic phase produced from the S-CB-L3 can be seen in Figure 12. The matrix is composed mainly of iron (~95 wt. %) based on the WDS point analysis in Table 4. The graphite flakes are randomly distributed, which is a typical appearance for the grey cast irons [44]. In the grey phase (marked as Ti-C in the BSE image), Ti, V, and C are concentrated. Other elements such as S, Cr, and Si have a low concentration.

## 4. Discussion

This section contains the discussion of the presented experimental results. 

### 4.1. Reduction of Oxides

**Figure 13:**
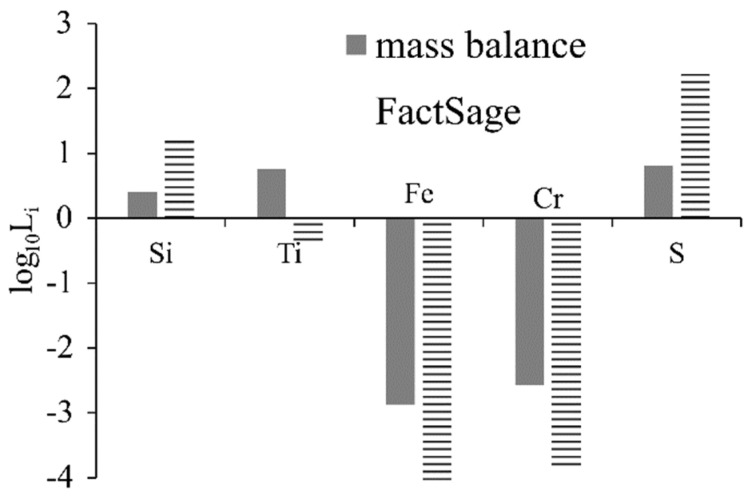
Distribution coefficient (L_i_) of Si, Ti, Fe, Cr, and S between mass balance and FactSage assumptions, in logarithmic scale.

Iron was almost completely reduced from the slags in the presence of carbon based on the results (XRF, XRD, and SEM/EDS) and the theoretical considerations. The iron phase was found to contain a low level of impurities (Table 4) while complex (Ti,V)C carbides were seen to precipitate. This was observed in other similar studies on the smelting-reduction of bauxite in graphite crucibles [9,45]. The distribution coefficient (L_i_) is described in Equation (1), in which (wt.% i) and [wt.% i] are the mass pct of element i in slag and metal, respectively and express the distribution of the elements between the slag and metal phases. The L_i_ was calculated according to the mass balance assumptions that were controlled to represent the experimental results and the FactSage predictions (Figure 4C). The distribution of the elements between the slag and metal can be schematically seen in Figure 13, in which species with positive log_10_L_i_ distributed in the slag phase and species with negative log_10_L_i_ are distributed in the metallic phase.

L_i_ = (wt.% i)/[wt.% i], (1)

Based on the theoretical results (Figure 4C), under carbon saturation conditions, Ti is concentrated in the metallic phase. Nevertheless, this was not confirmed through the experimental results (Table 4). Ti was distributed mainly in the slag phase. This contradiction might be related among other reasons to uncertainties in the databases used, as was pointed out in our earlier work [17]. Moreover, partial reduction of silica was predicted from the FactSage calculations and was also seen from the experimental results, although it is mainly concentrated in the slag phase. Cr was distributed mainly in the metallic phase while S was concentrated more in the slag phase. These characteristics are of interest for further utilization of pig iron. 

### 4.2. Aluminates in the Slags Produced in Different Crucibles

**Figure 14:**
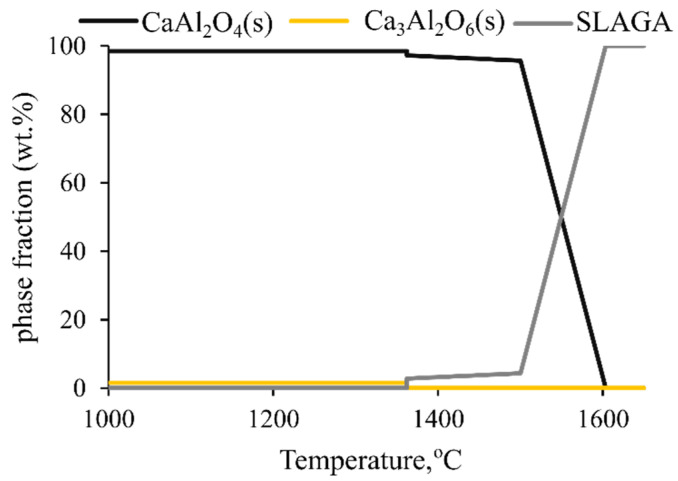
Predicted solidified slag phases for smelting in Al_2_O_3_ crucible as estimated with FactSage equilibrium calculations.

The studied mCaO/mAl_2_O_3_ ratios were resulted in phases that are considered leachable [9] (graphite crucibles), while increasing the lime addition enhances the formation of more C_5_A_3_. The formation of C_2_S and CT should be considered for optimum lime additions that depend on the ore characteristics. The dominant phase was the C_5_A_3_ and not the C_12_A_7,_ as was expected and will be discussed in the following section. Even though in terms of leachability both phases are equal, it is of interest to examine the effect of the process conditions in their stabilization. However, the stabilization of either C_5_A_3_ or C_12_A_7_ was not achieved when the Al_2_O_3_ crucible was used, most likely due to the dissolution of Al_2_O_3_ into the slag that might affect the slag phases [31]. In this case, main slag phases were the CA and CA_2_. Therefore, despite the higher $p_{O_{2}}$, the C_12_A_7_ was not the stable phase. As mentioned, the high Al_2_O_3_ content (Figure 7) and CA_2_ (Figure 10) indicate the dissolution of the crucible material in the melt. Nevertheless, the CaO/Al_2_O_3_ ratio of the obtained slag (S-A-RB, considering binary composition) indicate that the slag will be solidified in the crystallization area of CA. To further investigate the formation of CA_2_ FactSage calculations were done to predict the slag phases after solidification and the results are presented in Figure 14. The chemical composition of slag S-A-RB (excluding Fe-content) was considered for the calculations, while to make the calculations rather simple only the major components were selected. As can be seen, FactSage predicts that the solidified slag will be mainly composed of CA and to a lesser extent of C_3_A. 

**Figure 15:**
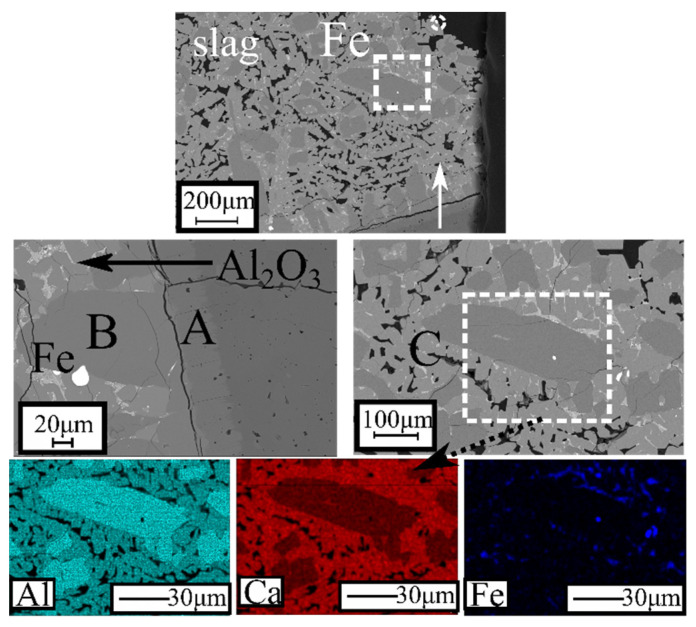
Backscattered electron images and X-ray mapping analysis of S-A-RB slag.

In correlation with the experimental results, the formation of CA_2_ was not predicted. To further understand the formation of CA_2_, the SEM analysis of slag S-A-RB is described in Figure 15. It seems that the crucible material has penetrated in the slag, creating an Al_2_O_3_-rich layer (marked A in Figure 15) while the phase B (appears as dark grey) seems to contain a higher concentration of alumina than CaO. Therefore, this phase is attributed to the CA_2_ phase that was mainly concentrated close to the Al_2_O_3_ crucible. Herein, the CA_2_ is distributed mainly close to the interphase with the Al_2_O_3_ crucible. This indicates that the dissolution is slow and as such it might be reasonable to assume that the dissolution is governed by kinetic effects that are not considered from the FactSage calculations. 

It is also seen that iron presents as metallic entrains in the slag phase while phase C might be attributed to the CA phase in correlation with the XRD analysis (Figure 10). The separation of the iron in a metallic phase can be achieved in a carbon-free atmosphere by the pre-reduction treatment. 

### 4.3. The Effect of Process Conditions

In the absence of humidity and/or oxygen, the formation of the C_12_A_7_ phase is not favored and the C_5_A_3_ phase will form instead [46]. The formation of C_12_A_7_ is favored in humid, ambient conditions. The C_12_A_7_ phase is in equilibrium with H_2_O above 950 °C and its stoichiometry in fully saturated conditions is defined as Ca_12_Al_14_O_32_(OH)_2_ [47,48]. The water presence is as hydroxyl anions in the crystal structure. In oxidizing atmosphere, C_12_A_7_ absorbs the excess of oxygen and it is stable even when $p_{O_{2}}$ is as low as 10^−8^ atm [49]. On the other hand C_12_A_7_ can be stabilized in the presence of a) other anions such as S, F^−^, Cl^−^ [50,51] or b) of SiO_2_/TiO_2_ [31,52]. Therefore, the existence of template anions can be satisfied from the reactants. The smelting of pure and less pure reactants in graphite crucibles (without lid) resulted in the formation of C_5_A_3_ and C_12_A_7_, respectively [31]. Recent studies are emphasizing the stabilization of C_12_A_7_ from C_2_^2−^ (from the graphite crucible) in reducing conditions ($p_{O_{2}}=10^{-16}\;\mathrm{atm}$) that can equilibrate the oxygen deficiency in its structure [53]. In addition, C_12_A_7_ might decompose to C_3_A and CA at temperatures higher than 1500 °C in a reducing, moisture-free, or inert atmosphere [54]. Nevertheless, the formation of C_12_A_7_ was not favored in the absence of O_2_/OH^−^ in the present study when the reduction was done in graphite crucible under dry Ar and estimated $p_{O_{2}}=6.54^{-16}\;\mathrm{atm}$. Therefore, the C_2_^2−^ contribution in the C_12_A_7_ stabilization seems to not be significant in the present study. According to the results of the current study, they might further support the theory that C_12_A_7_ might decompose to C_5_A_3_ in the absence of template anions due to its thermodynamic instability [21]. The decomposition of C_12_A_7_ into C_5_A_3_, C_3_A, and CA might happen upon cooling of the slag in dry-reducing atmospheres [20,31]. Nevertheless, the decomposition product will depend on the experimental conditions [20] and more work is required to identify whether decomposition occurs or not. In Table 5, the studied atmospheric conditions and obtain phases in the slags are summarized. The SiO_2_ presence may play a role in the stabilization of C_5_A_3_ as it may tie up the oxygen and thus slow down the formation of C_12_A_7_ [55]. Nevertheless, this was not observed in the present study since C_5_A_3_ would have been observed in all the obtained slags. An O_2_ containing atmosphere or small amount of H_2_O in an inert atmosphere can enhance the formation of C_12_A_7_ [22,48,49,56,57,58]. The results obtained in the present study supports the literature since C_12_A_7_ was the stable phase upon exposure in air (presence of O_2_ in open top furnace and estimated $p_{O_{2}}=4.38^{-2}\;\mathrm{atm}$) and in inert but moist atmosphere (5 vol.% H_2_O and estimated $p_{O_{2}}=5.63^{-16}\;\mathrm{atm}$). Although based on literature sources and the present study, the $p_{H_{2O}}\;$will affect the C_12_A_7_ stabilization, and the required levels of O_2_ or H_2_O that are necessary are not yet defined. Based on the thermodynamic predictions, the gas will contain H_2_/H_2_O/OH^−^ for the open top furnace and humid atmosphere. Therefore, it might be reasonable to assume that the stabilization of C_12_A_7_ is strongly affected by the presence of these species from the atmosphere. Frequently the literature reports the OH^−^ as the stabilizing agent. Nevertheless, it is currently unknown whether the exact mechanism should be addressed by the individual species.

It is proposed that the existence of atmospheric template anions affect in great extent the formation of C_12_A_7_ than other impurities_,_ and it is more stable under higher O_2,_ and H_2_O partial pressures compared to the inert atmosphere in which C_5_A_3_ is stable. Therefore, the stabilization mechanism that is suggested from the present study is the existence of O_2_/OH^−^ anions in the smelting atmosphere. Despite the fact that both phases are considered to be leachable in the Pedersen process [59], it is of scientific interest to investigate the effect of the smelting conditions in the formation of the several phases. Nonetheless, the complexity of the system makes it difficult to further conclude on the exact parameters that define the C_5_A_3_/C_12_A_7_ stabilization, and this will require more experimental and modelling work since the literature is scarce.

Increased cooling rates might result in the formation of less or even non-leachable phases such as C_3_A and C_2_AS [17,19]. Nevertheless, this was not observed in the present study since the C_5_A_3_ phase was the dominant phase under the applied slow and fast cooling rates. The SEM analysis (Figure 11) indicates that the microstructure of the S-CB-FC and the purity of the phases were more complex. A similar observation was made in our previous work related to the smelting of bauxite residue in which the increased cooling rate resulted in the formation of less pure phases and enhanced the formation of non-leachable phases [17]. This might affect the leaching efficiency due to the co-dissolution of Al_2_O_3_ and other impurities (mainly Si).

### 4.4. Alumina Recovery Evaluation 

As mentioned, the quantification of the phases with the Rietvelt method is considered semi-quantitative with respect of the limitations of the method. The results were cross-checked with the FactSage and HSC results. The HSC output analysis resulted in a residual error of 0.44 for the estimated phases and it was based on the XRF analysis of the slag. Table 6 presents typical results for the distribution of the phases in the final slag based on the Rietvelt, FactSage, and HSC results for the S-CB-open. The results were based on the composition of the final slag. Differences were observed for the phase amounts between the theoretical and experimental results. Both FactSage and HSC calculations are referred to equilibrium conditions which deviate from the experimental conditions, highlighting the limitations to predict and fully simulate a real case system. This can result in deviations between the theoretical predictions and experimental results in the product phases and their participation in the final slag. A typical example is that the formation of CA was not predicted from FactSage and HSC while its participation in the sample was evidenced through XRD and EPMA results. Moreover, limitations in the thermodynamic data used is another aspect that needs to be considered.

In addition, possible inhomogeneities among the samples can also deviate the results. As an example, a change in the TiO_2_, considering the range of deviation observed from the repeated experiments, can result in approximately a 10% difference in the percentage of CaO.TiO_2_ in the final slag. The distribution of C_12_A_7_ and C_2_S was crosschecked within an acceptable range considering the aspects discussed earlier. The participation of CT was however less satisfying and further evaluation in the properties of this phase, the thermodynamic data, and sample refinement might be necessary. In general, less deviation was observed for the main calcium aluminate phase between the FactSage, HSC, and Rietveld results. 

The semi-quantitative Rietvelt analysis indicates the distribution of the stable oxides between the detected phases. The lack of C_2_S (S-A-SR and S-CB-L2) and the formation of C_2_AS (S-A-CB, S-CB-L2) foreshadows possible Si contamination and retardation in the alumina recovery. The presence of SiO_2_ as a free oxide should be controlled. The formation of C_2_S is believed to prevent the dissolution of SiO_2_ and the contamination into the leaching solution, if all the input SiO_2_ is distributed in the C_2_S phase. In addition, the C_2_AS is a phase that retards the alumina recovery. If all Al is in leachable phases, then more than 90% of Al recovery can be achieved [60]. As such, the samples with higher mCaO/mAl_2_O_3_ (S-CB-L3, S-CB-H2O, and S-CB-open), believed to result in high alumina recoveries since the input alumina, participated in the leachable phases. Nevertheless, the exact yield of alumina will be further defined from the leaching parameters and kinetics.

Finally, the smelting of reduced bauxite resulted in slag with proper characteristics (identical to the slag from calcined bauxite) and this might be important as the pre-reduction of bauxite can decrease the CO_2_ gas emissions from the smelting furnace while also increasing its capacity. The control in slag chemistry and smelting conditions (i.e., cooling rate) can ensure the formation of slags that could be used for Al_2_O_3_ recovery and in this way make use of lower grade bauxites or other Al-sources that are not considered proper for the Bayer process or that are disposed of.

## 5. Conclusions

The smelting reduction of reduced and calcined bauxite samples were carried out and resulted in the formation of pig irons and slags. The following concluding remarks can be made:

- Iron was reduced and separated from the slags in the presence of carbon regardless of the characteristics of the feed material (reduced and calcined bauxite). This was further confirmed from the thermodynamic evaluations. Iron was separated in the form of pig iron that was found to contain low level of impurities such as Si, S, and Ti, and more specifically:
  - Cr was distributed in the metallic phase;
  - Ti was precipitated in complex (Ti,V)C carbide particles;
  - Si and Ti were mainly distributed in the slag;
- In the absence of carbon (alumina crucible), iron was separated from the slag for the reduced bauxite, and this offers promising perspective for a process with reduced CO_2_ emissions;
- The distribution of the major species and phases in the solidified slags was crosschecked between the thermodynamic predictions and experimental results. Differences for the minor phases and quantification aspects require further evaluation of the thermodynamic data and limitations;
- Slags with alumina containing leachable phases were formed in graphite crucible while increasing the lime addition resulted in the formation of C_5_A_3,_ which is considered a highly leachable phase. As such, high alumina recoveries can be assumed according to the phase quantification;
- The dissolution of the alumina crucible into the slags and the provided atmospheric conditions (absence of C, higher $p_{O_{2}}$) result in changes in the product phases of the slags as compared to the graphite crucible. The slags contain both leachable (CA) and non-leachable (C_2_AS, CA_2_) phases and this is expected to retard the alumina recovery;
- The stabilization of C_5_A_3_/C_12_A_7_ was seen to be affected mainly from the atmospheric conditions that include the presence of higher partial pressures of O_2_/OH^−^.

## Figures and Tables

**Table 1:** Experimental conditions.

Test Code	Crucible	mCaO/mAl_2_O_3_	Holding Time (min)	Average Cooling Rate ^a^ (°C/min)	Atmospheric Conditions during Smelting	$\mathrm{Estimated}\;p_{O_{2}}{\;}^{b}$ (atm)
S-CB-T1	Graphite	0.88	60	10	Ar, Reducing in absence of O_2_, presence of C	
S-CB-T2/L1	Graphite	0.88	45	10	Ar, Reducing in absence of O_2_, presence of C	
S-CB-L2	Graphite	0.95	45	10	Ar, Reducing in absence of O_2_, presence of C	
S-CB-L3	Graphite	1.12	45	10	Ar, Reducing in absence of O_2_, presence of C	5.62 × 10^−16^
S-RB	Graphite	1.12	45	10	Ar, Reducing in absence of O_2_, presence of C	
S-CB-FC	Graphite	1.12	45	26	Ar, Reducing in absence of O_2_, presence of C	
S-CB-open	Graphite	1.12	45	10	Reducing in presence of O_2_ and C, exposure to air	4.38 × 10^−2^
S-CB-H_2_O	Graphite	1.12	45	10	Ar- (5 vol.%) H_2_O	5.63 × 10^−16^
S-A-CB	Alumina	1.12	45	10	Ar, absence of C	0.17
S-A-RB	Alumina	1.12	45	10	Ar, absence of C	0.17

^a^ to 1000 °C, ^b^ based on FactSage calculations.

**Table 2:** Chemical composition (wt.%) of the raw materials as measured by XRF with the calculated st. deviation and Lime as received.

	Al_2_O_3_	Fe_2_O_3_ * or Fe **	SiO_2_	TiO_2_	CaO	Cr_2_O_3_	MgO	CaCO_3_
wt.%								
CB	65.1 ± 0.4	25.6 ± 0.3 *	2.00 ± 0.1	2.80 ± 0.1	4.10 ± 0.1	0.101 ± 0.1	0.00	0.00
RB	71.1 ± 0.6	18.6 ± 0.2 **	2.31 ± 0.4	3.21 ± 0.1	4.42 ± 0.1	0.202 ± 0.1	0.00	0.00
Lime	0.3	0.1	0.5	0.1	96.6	0.0	0.7	1.7

* Fe_2_O_3_ in CB, ** Fe in RB.

**Table 3:** Semi-quantitative Rietvelt analysis of selected slag samples.

Mineral Formula	S-A-CB	S-A-SR	S-CB-L1	S-CB-L2	S-CB-L3	S-CB-H_2_O	S-CB-Open	ICCD Number, Reference
CA	72	76	64	55	-	42	18	04-013-0779, [32]
CA_2_	10	17	-	-	-	-	-	00-023-1037, [33]
CT	3	2	4	5	5	6	6	04-015-4851, [34]
C_2_AS	3	5	-	2	-	-	-	04-014-4683, [35]
C_3_A	-	-	30	38	-	-	-	00-006-0495, [36]
C_5_A_3_	-	-	-	-	92	-	-	04-007-8643, [37]
C_12_A_7_	-	-	-	-	-	50	72	00-009-0413, [38]
C_2_S *	3	-	-	-	-	-	-	00-033-0302, [39]
C_2_S **	4	-	2	-	3	3	4	04-012-6734, [40]
FeO	2	-	-	-	-	-	-	04-011-7345, [41]
Fe_3_O_4_	3	-	-	-	-	-	-	04-015-9120, [42]
Rwp	8.8	11.8	6	6.1	9.5	9	11.8	-

* Larnite monoclinic, ** Calcio olivine orthorhombic.

**Table 4:** Averaged chemical composition of slag phases for the samples S-CB-L3, S-CB-FC and S-CB-open based on EDS point analysis and metal phases based on EPMA (WDS) analysis.

Phases	Suggested Phase	Slags							
S-CB-L3 (Atomic %)									
		Al	Ca	O	Mg	Si	Ti	V	C
A-L3	C_5_A_3_	26.5	27.1	45.3	0.52	0.62	-	-	-
B-L3	CT	12.5	24.8	50.4	0.62	2.22	9.61	-	-
C-L3	TiC	-	0.9	-	-	0.7	47.1	13.1	37.2
S-CB-FC (Atomic %)									
A-FC	C_5_A_3_	26.8	26.4	46.1	0.33	0.71	-	-	-
B-FC	C_2_AS	16.5	23.2	52.7	1.12	6.21	0.72	-	-
C-FC	CT/C_2_AS	26.9	27.2	46.9	0.91	2.31	5.82	-	-
D-FC	C_5_A_3_/C_2_AS	20.6	43.8	30.9	0.52	2.31	1.83	-	-
S-CB-open (Atomic %)									
A-open	C_12_A_7_	34.4	19.9	45.7	-	-	-	-	-
B-open	CA/C_2_S	18.7	31.6	44.5	0.83	3.61	0.82	-	-
C-open	CT/CA	17.5	24.2	44.8	0.72	0.91	8.44	-	-
Sample	Metals *								
		Fe	Ti	V	Cr	Si	S	C	
S-CB-L3		99.5	0.03	0.21	0.31	0.01	0.01		
S-RB		99.2	0.03	0.21	0.50	0.01	0.01		

* averaged from 5 parallel measurements.

**Table 5:** Impact of atmospheric conditions during smelting and crucible material on the obtained slag phases.

Conditions during Smelting	Crucible Material	Obtained Slag Phase
Exposure in Air	Graphite	C_12_A_7_
Inert dry Ar	Graphite	C_5_A_3_
Ar-H_2_O	Graphite	C_12_A_7_
Absence of C	Al_2_O_3_	Neither C_12_A_7_ nor C_5_A_3_

**Table 6:** Comparison of the phase distribution between HSC, FactSage, and Rietvelt results for the S-CB-open.

Mineral Formula	Rietvelt	FactSage	HSC
12CaO.7Al_2_O_3_	72	86	87
CaO.Al_2_O_3_	18	-	-
CaO.3Al_2_O_3_	-	5	4
CaO.TiO_2_	6	-	4
Ca_3_Ti_2_O_7_ (ss)	-	4	-
2CaO.SiO_2_	4	5	5

## Data Availability

Not applicable.
